# Erectile dysfunction in a cohort of HIV-infected male patients

**DOI:** 10.1186/1471-2334-14-S7-P31

**Published:** 2014-10-15

**Authors:** Narcis Chirca, Anca Streinu-Cercel, Viorel Jinga, Adrian Streinu-Cercel

**Affiliations:** 1 Urology Department, Clinical Urology Hospital “Prof. Dr. Th Burghele” Bucharest, Romania; 2 Carol Davila University of Medicine and Pharmacy, Bucharest, Romania; 3 National Institute for Infectious Diseases "Prof. Dr. Matei Balş", Bucharest, Romania

## Background

The advent of antiretroviral therapy has transformed HIV infection into a manageable chronic disease, where improving the quality of life of HIV-infected patients has become one of the main focus points for physicians. The aim of our study was to evaluate the prevalence of erectile dysfunction and testosterone deficiency in a cohort of HIV-infected patients monitored in the National Institute for Infectious Diseases "Prof. Dr. Matei Balş" Bucharest, from May 2014 to June 2014.

## Methods

We evaluated a cohort of HIV-infected male patients. They completed a questionnaire to evaluate erectile dysfunction, based on the International Index of Erectile Function – IIEF (maximum score 30 for questions 1,2,3,4,5,15). Total testosterone was dosed in a subset of patients reporting erectile dysfunction (mild and moderate or severe) considering normal values >10 nmol/L. Five patients refused the test.

## Results

42 patients completed the questionnaire; they had ages between 23 and 69 years (mean age 36.8), 38 of them (90.4%) receiving antiretroviral therapy. 15 patients had a degree of erectile dysfunction (35.7% of total) and 2 patients had had no sexual activity in the last month: 12 patients had mild erectile dysfunction (score between 19-24), 2 patients had mild to moderate erectile dysfunction (score between 13-18), 1 patient had moderate erectile dysfunction (score between 7-12). Total testosterone was tested for 3 patients, 5 refused the test; all tests revealed normal values (>20 nmol/L).

## Conclusion

This study showed that erectile dysfunction is highly prevalent, 35.7% of HIV-infected male patients have reported erectile problems.

